# Natural Variation of Cold Deacclimation Correlates with Variation of Cold-Acclimation of the Plastid Antioxidant System in *Arabidopsis thaliana* Accessions

**DOI:** 10.3389/fpls.2016.00305

**Published:** 2016-03-17

**Authors:** Ilona Juszczak, Jelena Cvetkovic, Ellen Zuther, Dirk K. Hincha, Margarete Baier

**Affiliations:** ^1^Plant Physiology, Dahlem Center of Plant Sciences, Free University of BerlinBerlin, Germany; ^2^Molecular Physiology, Institute of Molecular Physiology and Biotechnology of Plants, University of BonnBonn, Germany; ^3^Max-Planck-Institute of Molecular Plant PhysiologyPotsdam, Germany

**Keywords:** cold, Arabidopsis, plastid antioxidant system, reactive oxygen species, natural variation

## Abstract

Temperature variations impact on the balance between photosynthetic electron transport and electron-consuming assimilation reactions and transiently increase generation of reactive oxygen species (ROS). Previous studies demonstrated that the expression of C-repeat binding factors (CBFs), which activate cold acclimation reactions, respond to chloroplast ROS signals and that cold deacclimation is partly halted for days after the transfer of acclimated plants to optimal growth conditions in four Arabidopsis accessions from cold-continental habitats. We hypothesized that these accessions differ from others in the regulation of the plastid antioxidant system (PAS). In the present study, we compared the expression intensity of the 12 most prominent PAS genes for peroxidases, superoxide dismutase and low molecular weight antioxidant regenerating enzymes in 10 Arabidopsis accessions with regulation of *CBF* and *COR* (cold regulated genes) transcript levels and cold-regulated metabolite levels prior to cold, after 2 week long cold acclimation and during the first 3 days of deacclimation. In the accessions with prolonged activation of cold responses, by trend, weaker induction of various cold-inducible PAS genes and stronger decreases in the expression of negatively cold-regulated PAS genes were observed. Low PAS gene expression delayed the post-cold decrease in H_2_O_2_ levels after transfer of the plants from cold to optimal growth conditions. We conclude that weaker expression of various PAS genes in the cold is an adapted strategy of the Arabidopsis accessions N14, N13, Ms-0, and Kas-1 to avoid full inactivation of cold-responses in the first days after the end of the cold period.

## Introduction

Photosynthesis dominates plant energy metabolism. However, it also bears a strong risk for generation of reactive oxygen species (ROS) under instable environmental conditions, such as variable temperature or light intensity (Baier and Dietz, 1999; Ensminger et al., 2006). Transfer of energy from excited pigments or transfer of electrons from the photosynthetic electron transport chain to oxygen leads to the formation of singlet oxygen (^1^O_2_), superoxide anions (O${}_{2}^{-}$), hydroxyl radicals (HO^•^) and hydrogen peroxide (H_2_O_2_; Foyer et al., 1994; Asada, 2000). Excess amounts of ROS damage enzymes and structural cell components (Baier and Dietz, 1999). In parallel and already at lower doses, ROS drive beneficial signal transduction cascades and stress acclimation reactions (Desikan et al., 2001; Baxter et al., 2014).

The plastid antioxidant system (PAS) counteracts production and accumulation of ROS directly inside chloroplasts (Asada, 2000; Baier and Dietz, 2005). The key enzymes are Cu/Zn superoxide dismutase (Csd2), stromal and thylakoid-bound ascorbate peroxidases (sAPx and tAPx), 2-Cys peroxiredoxins (2CPA and 2CPB), peroxiredoxin-II-E (PrxIIE), peroxiredoxin Q (PrxQ), glutathione peroxidases (GPx), glutathione reductase (GR), and mono- and dehydroascorbate reductases (MDHAR and DHAR). These enzymes are interconnected by low molecular weight antioxidants such as ascorbate and glutathione, and redox proteins, such as thioredoxins and glutaredoxins (Baier et al., 2010). Csd2 converts O${}_{2}^{-}$ in the vicinity of thylakoids into H_2_O_2_ and O_2_ (Bowler et al., 1994; Rizhsky et al., 2003). Ascorbate peroxidases detoxify H_2_O_2_ at the expense of ascorbate (Asada, 2000). Peroxiredoxins and glutathione peroxidases reduce H_2_O_2_ and alkyl hydroperoxides (Rouhier and Jacquot, 2005; Dietz et al., 2006). They use small thiol proteins, such as thioredoxins and glutaredoxins, as co-substrates. MDHAR, DHAR, and GR regenerate ascorbate and glutathione (Asada, 2000). Most of these enzymes are located in the chloroplast stroma or are loosely attached to the thylakoids (Asada, 2000; Dietz et al., 2006), while PrxQ acts inside the thylakoids (Petersson et al., 2006) and tAPx is anchored in the thylakoids by a C-terminal transmembrane helix (Miyake and Asada, 1992).

A sudden drop in temperature causes transient redox imbalances and increased ROS production especially inside chloroplasts (Huner et al., 1998; Ensminger et al., 2006). Low temperatures slow down biochemical reactions more strongly than electron transport processes (Ensminger et al., 2006). As a result, photosystem II excitation pressure and the reduction state of the chloroplast stroma increase. In contrast to e.g., light-induced photooxidative stress, expression of photosynthesis-associated genes (PhaGs) remains active under these conditions (Huner et al., 1998; Strand et al., 1999; Savitch et al., 2001). CO_2_-fixation often even slightly increases to support osmolyte production (Strand et al., 1997; Byun et al., 2014) and re-stabilization of photostasis (Ensminger et al., 2006).

Cold responses are mediated by specific signal transduction pathways (Thomashow, 1999; Fowler and Thomashow, 2002). The most prominent is the CBF (C-repeat binding factor)-regulon (Thomashow, 1999). It drives *COR*-gene (cold regulated genes) expression and osmolyte accumulation. Chloroplasts and especially photosynthesis have been proposed to be the main cold sensors (Ensminger et al., 2006). Signal transduction takes place via CBF1-mediated induction of DELLA-protein expression and activation of gibberellin catabolism and arrests growth and development (Achard et al., 2008). Acclimation re-establishes photostasis and antagonizes ROS accumulation (Strand et al., 1999; Ensminger et al., 2006; Juszczak et al., 2012).

At the end of the cold-period, plants quickly have to reorganize their metabolism, activate growth, and produce new leaves to compete successfully with neighboring plants for space and light. Under laboratory conditions, about half of the genes that are regulated by cold, are readjusted within 24 h after the cold period in *Arabidopsis thaliana* Col-0 (Byun et al., 2014). In parallel, the cell division and elongation rates increase from an almost complete arrest to levels close to pre-cold ones (Byun et al., 2014). Compared to acclimation, the process of deacclimation has been much less investigated. Our recent study (Zuther et al., 2015) demonstrated in a series of 10 Arabidopsis accessions that it is, like acclimation (Hannah et al., 2006), genetically determined. Expression of CBF- and COR-genes and biosynthesis of osmolytes quickly decline in all accessions within the first 24 h. However, after 3 days at ambient temperature, the levels of cold induced osmolytes and expression of CBF-controlled genes, especially that of COR78 (RD29A) and GolS3 (encoding galactinol synthase), are still higher in the accessions N14, N13, Ms-0, Kas-1, and (to a lesser extent) WS than in Col-0, Van-0, Sah-0, Can-0, and C24. Also the re-setting of metabolite and transcript levels is halted and osmolyte and transcript levels remain slightly elevated for 3 days (Zuther et al., 2015). Activation of cold-responsive genes and metabolite synthesis are cost-intensive (Browse and Lange, 2004). Consequently, the cold acclimation response can be assumed to be actively maintained at slightly elevated levels (as compared to pre-cold levels) and provide higher freezing tolerance (as indicated by the LT_50_; Zuther et al., 2015; Supplementary Table 1), when cold acclimated plants are transferred back to optimal growth conditions.

Information about the control of CBF expression at ambient temperature is available from analysis of transgenics and mutants: *CBF1* expression decreases in response to *tAPx* silencing (Maruta et al., 2012). *Vice versa, CBF1* is more highly expressed in *gun5-1* and *cch* mutants, in which chlorophyll-biosynthesis, chloroplast maturation, and development of the thylakoid membrane are impaired (Kindgren et al., 2015). While the latter approach did not impact on regulation of downstream genes of the CBF-regulon, silencing of *tAPx* did.

The PAS is a network of enzymes with overlapping functions (Asada, 2000; Baier et al., 2010). Based on the observation by Maruta et al. (2012) that CBF1 and the CBF-regulon can be induced in response to insufficient plastid peroxidase activity, we analyzed the same series of 10 *Arabidopsis thaliana* accessions as in Zuther et al. (2015) before (NA) and 0 (ACC), 1, 2, and 3 days after a 2-week period at 4°C (DEACC1–DEACC3) for regulation of the most prominent genes for PAS enzymes. All data were arranged along a gradient, which reflects the extent of frost tolerance acquired by the respective accessions during 2 weeks at 4°C (LT_50_; Supplementary Table 1) like in Zuther et al. (2015). We show that the same accessions, which maintain part of their cold-responses have a delayed shift in the ROS signature during deacclimation and lower transcript abundance of genes encoding specific chloroplast antioxidant enzymes after acclimation.

## Materials and methods

### Plant material and growth conditions

*Arabidopsis thaliana* accessions of the NASC (stock numbers: N1264 and N906) and INRA stock collections (stock numbers: 84AV, 93AV, 161AV, 163AV, 186AV, 233AV, 266AV, and 267AV) were grown for 42 days on soil in a day/night regime (16 h day; 8 h night) at 20°C day temperature and 18°C night temperature and 200 μmol quanta m^−2^ s^−1^ as described in Hannah et al. (2006). They were also transferred to a 4°C cold chamber for 2 weeks and illuminated at 90 μmol quanta m^−2^ s^−1^ in the same day/night pattern like in Hannah et al. (2006). Afterwards they were transferred back to the 20/18°C conditions for deacclimation. The plant material is identical to or was harvested side by side with that used in Zuther et al. (2015) at an age of 42 days (NA), after 14 days cold acclimation (ACC), and after 1, 2, and 3 days of deacclimation (DEACC1, DEACC2, DEACC3) 6–7 h after onset of light. The leaf rosettes were either frozen immediately in liquid nitrogen for metabolite of transcript abundance analysis or directly stained for H_2_O_2_ or radicals.

### qRT-PCR analyses

Total RNA was extracted from frozen plant material. For each of the three biological replicates, which were grown at different times of the year (spring, summer, and autumn), material from five plants was pooled. Extraction was performed with either Trizol reagent (Invitrogen) or tissue lysis buffer (100 mM Tris-Cl pH 8.5–9.0; 25 mM EDTA, 25 mM EGTA, 2% (w/v) SDS, 100 mM 2-mercaptoethanol) supplemented with 1 volume phenol, 1 volume chloroform and 1/24 volume isoamylalcohol. RNA quality control, DNase digestion, first strand DNA synthesis, and cDNA quality controls were performed as described in Juszczak et al. (2012). Quantitative PCR was performed with an ABI PRISM7900 HT 384-well plate Sequence Detection System (Applied Biosystems, Darmstadt, Germany). Each sample contained 2.5 μl 2x SYBR Green Master Mix (Fast Power SYBR Green, Applied Biosystems), 0.5 μl cDNA (5-fold diluted) and 2 μl of 0.5 μM primers (Supplementary Table 2). The C_t_ values of the genes of interest were normalized by subtracting the mean C_*t*_ of the four reference genes (Act2, GAPDH, EXPRS, PDF2; Supplementary Table 1). All normalized expression values are listed in Supplementary Figure 1.

### Histochemical analyses of superoxide and hydrogen peroxide accumulation

Histochemical analyses of radical (R^•^) and hydrogen peroxide (H_2_O_2_) levels were performed according to Juszczak and Baier (2014) with nitroblue tetrazolium [NBT; 1 mg/ml NBT in 10 mM NaN_3_, 8% (m/v) NaCl, 0.2% (w/v) KCl, 1.44%(w/v) Na_2_HPO_4_ and 0.24% (w/v) KH_2_PO_4_; pH 7.4] and 3,3-diaminobenzidine [DAB; 1 mg/ml DAB in 8% (m/v) NaCl, 0.2% (w/v) KCl, 1.44%(w/v) Na_2_HPO_4_ and 0.24% (w/v) KH_2_PO_4_; pH 7.4]. All leaves were transferred immediately into the staining solution upon harvest and infiltrated at very low light intensity with the staining solutions. The staining was continued under soft shaking in darkness. Background staining was removed in a 1:1:3 mixture of acetic acid, glycerol and ethanol at 60–80°C. The staining intensity was quantified on digital images after 32-bit gray scale transformation by using the “mean gray value” analysis tool of the ImageJ software package (Schneider et al., 2012).

### Determination of chlorophyll and anthocyanin contents

Total chlorophyll contents and chlorophyll-a/chlorophyll-b ratios (Chl a/b) were determined spectrophotometrically in acetone extracts of 10–20 mg of plant material according to Porra et al. (1989). Anthocyanin contents were determined spectrophotometrically in acidic methanol extracts as described by Mancinelli et al. (1975).

### Determination of ascorbate contents and the redox state of the ascorbate pool

The levels of reduced and total ascorbate were quantified enzymatically with ascorbate oxidase as described in Baier et al. (2000) from the difference of the sample absorptions prior and 30 s after addition of ascorbate oxidase. The amount of enzyme was optimized that it is sufficient to oxidize all ascorbate in the sample within less than 20 s. The recovery rate was calculated from the comparison of extracts and reference samples, for which plant material was mixed with ascorbate standards prior to the extraction. Calibration was performed with dilution series for ascorbate and (9+1), (8+2), and (7+3) mixes of ascorbate and dehydroascrobate.

### Statistical analyses and additional data

Tukey *Post-hoc* tests, Students *t*-Test, Pearson correlation analysis (r_*P*_) and regression analysis were performed with crude data sets and cumulated data (means and differences) using SPSS22. Data on proline (Pro), glucose (Glc), fructose (Fru), sucrose (Suc), and raffinose (Raf) levels and the LT_50_ values for the various Arabidopsis accession after acclimation were taken from the analysis of Zuther et al. (2015), which was performed with the same or parallel grown plant material. The LT_50_ was determined using an electrolyte leakage assay on detached leaves frozen to various temperatures between −1°C and −25°C. Spearman rank order correlation analysis (as depicted in **Figures 7, 8** and in Supplementary Figures 3, 4) was performed in R using the command *rcorr* from the package *Hmisc*. *P*-values were set to 0.05.

## Results

Arabidopsis almost fully arrests its growth when it is transferred to 4°C (Scott et al., 2004). Consequently we compared the data obtained for the 10 investigated accessions after the cold treatment (ACC and DEACC) with the pre-cold status (NA) of the accessions, to avoid developmental effects, such as by comparing 2 week long cold-treated and growth arrested plants with untreated ones of the same age (8 weeks).

### Chlorophyll levels upon long-term cold stress

Chloroplasts are one of the main integration sites of acclimation processes to various types of environmental stress (Crosatti et al., 2013). Limitations in the recovery of plastid function, such as mutations in chlorophyll biosynthesis and chloroplast translation (Kindgren et al., 2015), and excess ROS (Maruta et al., 2010; Kurepin et al., 2013) induce *CBF* expression. As indicators for the chloroplast status, the chlorophyll levels (Chl a+b) and the chlorophyll a/b ratios (Chl a/b ratio) were compared in 10 Arabidopsis accessions.

**Figure 1:**
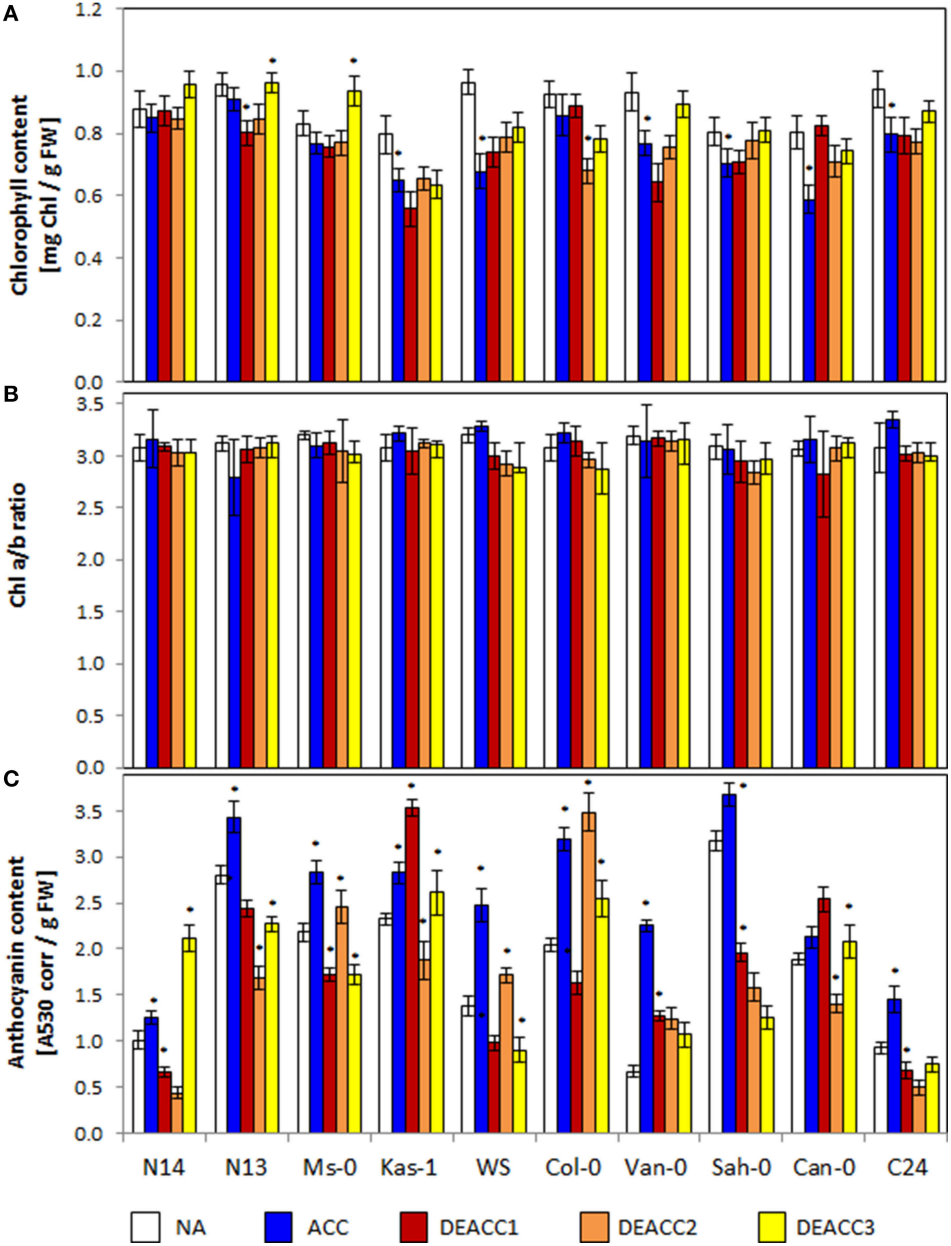
**Chlorophyll contents (A), Chl a/b ratio (B), and anthocyanin contents (C) in the rosettes of the 10 investigated Arabidopsis accessions per g freshweight (FW)**. Plants were harvested before (NA) or after (ACC) 14 days of cold acclimation at 4°C and after 1, 2, or 3 days of deacclimation (DEACC1, DEACC2, and DEACC3) at 20/18°C day/night temperatures. Accessions were ordered from the lowest LT_50_ after cold acclimation on the left to the highest on the right. Bars represent means ± standard deviation (*n* = 9). Statistically significant changes (Tukey *post-hoc*, Student *t*-Test; *P* < 0.1) relative to the previous day are labeled with an asterisk.

The total chlorophyll content serves as an indicator for the overall availability of photoreaction centers and chlorophyll-binding antenna proteins, while the Chl a/b ratio provides information on the compositions of the photosystems, e.g., on the antenna size (Ballottari et al., 2007). For additional comparison of the cold and deacclimation responses within the accessions, the ACC and DEACC values depicted in Figure 1 were normalized on the NA levels of the respective accession and depicted in Supplementary Figure 5. Regression analysis did not show any general trend relative to the LT_50_ value (data not shown). However, 14 days of cold treatment decreased the chlorophyll levels in Kas-1, WS, Van-0, Sah-0, Can-0, and C24 significantly, but not in N14, N13, and Ms-0 (Figure 1A). N14, N13, and Ms-0 over-compensated the chlorophyll levels during the deacclimation phase (DEACC plants; Figure 1A; Supplementary Figure 5A) excluding *CBF*-regulation due to limitations in the thylakoid recovery potential (Kindgren et al., 2015).

The Chl a/b ratio was not significantly changed in any accession during cold acclimation and it was only decreased below starting levels during deacclimation in WS and Col-0 (Figure 1B; Supplementary Figure 5B). These data show that the treatments were mild enough not to exhaust regulation of the plastid encoded photoreaction centers and the mainly nuclear encoded antenna proteins.

### Anthocyanin accumulation

Anthocyanin (Antho) levels combine information on excess excitation (Chalker-Scott, 1999), cold responses (Catalá et al., 2011) and carbohydrate availability (Laby et al., 2000). Anthocyanins accumulate preferentially in the epidermis and shield the photoreaction centers in the mesophyll from red, blue, and UV-light (Leyva et al., 1995). The anthocyanin contents strongly varied in the tested accessions prior to the cold treatment (NA), and generally increased during cold acclimation (ACC; Figure 1C). The lowest levels prior to the cold-treatment were observed in N14, Van-0, and C24, the smallest increases in N14, Sah-0, and Can-0 and the strongest increases in Van-0, WS, and Col-0. During the course of deacclimation, the anthocyanin levels quickly decreased in most genotypes. In Kas-1 and Can-0 they increased on the first day of deacclimation (DEACC1), before they also declined. In Ms-0, WS, and Col-0, they increased transiently on the second day of deacclimation (DEACC2), in N14, N13, Kas-1, C24, and Can-0 on the third day of deacclimation (DEACC3), but do not indicate a trend relative to the freezing tolerance of the accessions.

### Reactive oxygen species production upon long-term cold stress

**Figure 2:**
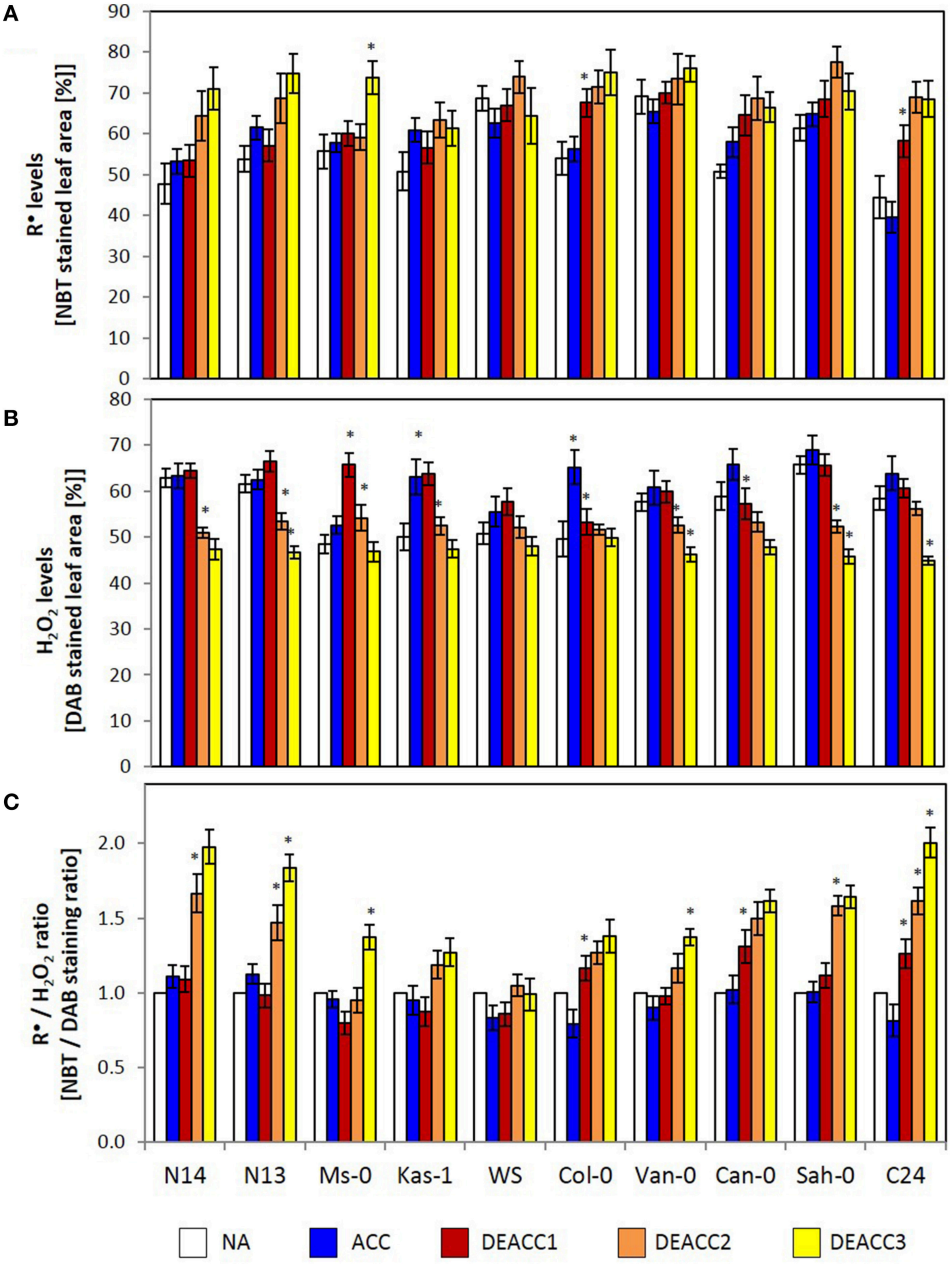
**The relative R^•^-level (determined as percentage of NBT stained leaf area) (A), the relative H_2_O_2_-level (determined as percentage of DAB stained leaf area) (B) and the R^•^/H_2_O_2_ ratio (as determined based on the NBT and DAB staining data) (C) in the rosettes of the 10 investigated Arabidopsis accessions**. Plants were harvested before (NA) or after (ACC) 14 days of cold acclimation at 4°C and after 1, 2, or 3 days of deacclimation (DEACC1, DEACC2, and DEACC3) at 20/18°C day/night temperatures. Accessions were ordered from the lowest LT_50_ after cold acclimation on the left to the highest on the right. Bars represent means ± standard derivation (*n* = 5). Statistically significant changes (Tukey *post-hoc*, Student *t*-Test; *P* < 0.1) relative to the previous day are labeled with an asterisk.

Singlet oxygen, reactive radicals and hydrogen peroxide accumulate, if the PAS does not fully counteract photosynthetic imbalances. Here, staining with nitroblue tetrazolium (NBT) and 3,3′-diaminobenzidine (DAB) was applied to detect changes in the ROS ratios. NBT staining mainly responds to radicals (R^•^), such as O${}_{2}^{-}$. In all accessions the staining intensity was similar before and after 14 days at 4°C, demonstrating acclimation of the antioxidant system and of photosynthesis (Figure 2A). Differences were observed in the post-cold period: The NBT staining intensity showed the highest increase in the accessions with the lowest LT_50_ (N14, N13, and Ms-0; Zuther et al., 2015) and in C24, while Kas-1 showed the smallest increase.

DAB-staining mainly records H_2_O_2_ levels. The staining intensities did not differ much between the accessions before the cold treatment (Figure 2B). In the accessions with lowest and highest LT_50_, it decreased during deacclimation to levels lower (at DEACC3) than those prior to the cold-treatment (NA). In N14, N13, Kas-1, Van-0, and Sah-0 stronger decreases were observed on the second day after the cold period than on the first day, indicating that H_2_O_2_ detoxification was delayed.

As an indicator for the efficiency of coupling H_2_O_2_ detoxification to radical accumulation, the NBT staining intensity (NBT%) was analyzed relative to the DAB staining intensity (DAB%) and normalized to the starting values (NA values; Figure 2C). In the accessions with lowest LT_50_, Can-0, Sah-0, and C24, the R^•^/H_2_O_2_-ratio increased on DEACC1. In all other accessions, it increased from DEACC2 onwards, except WS, in which generally the smallest changes in the ROS-signature were observed and the R^•^/H_2_O_2_-ratio was similar to non-treated plants after DEACC1.

### Activation level of ROS signaling marker genes

**Figure 3:**
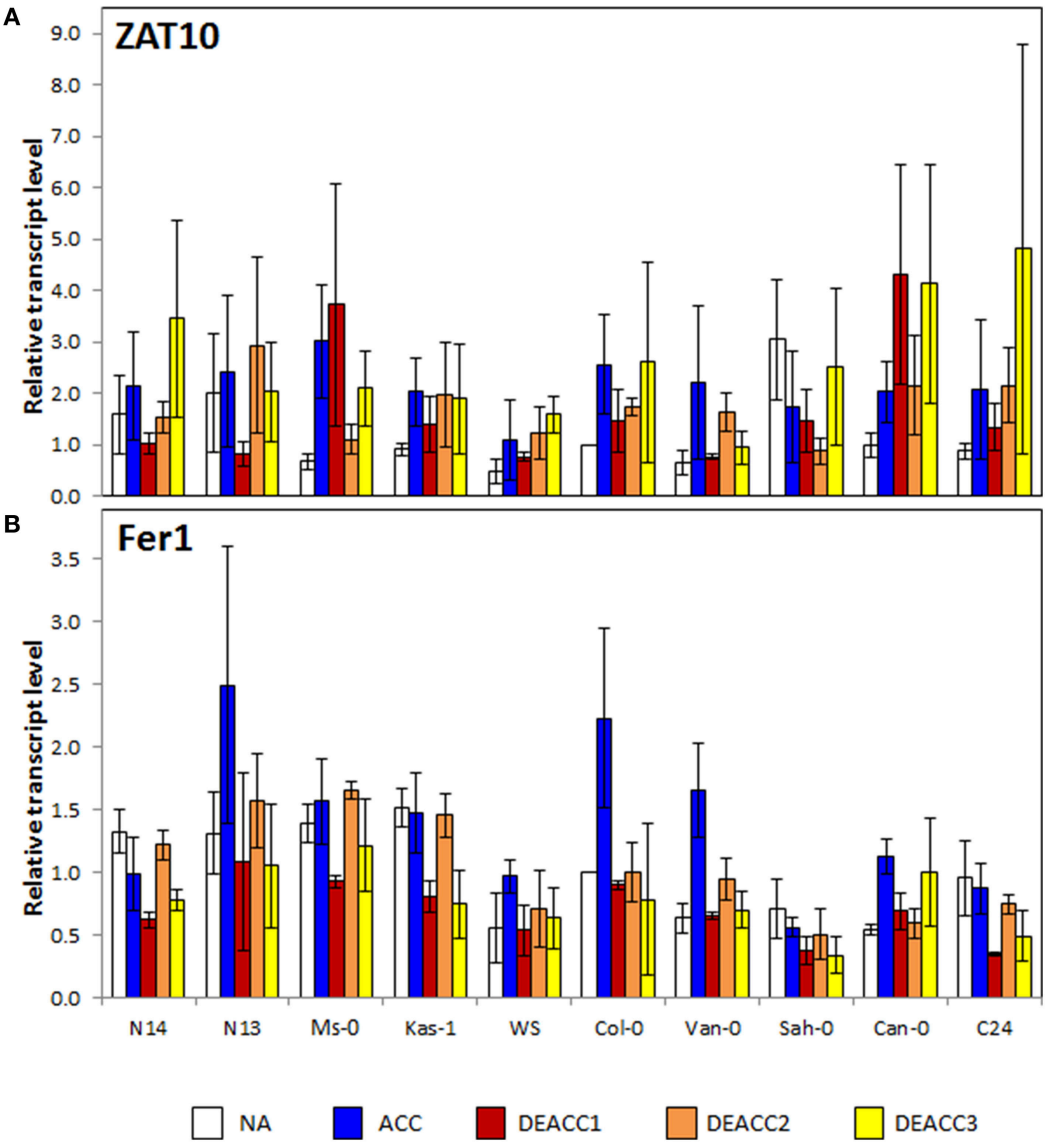
**Transcript level of *ZAT10* (A), and *Fer1* (B) in the rosettes of the 10 investigated Arabidopsis accessions relative to the transcript level in Col-0 prior to the cold treatment**. Plants were harvested before (NA) or after (ACC) 14 days of cold acclimation at 4°C and after 1, 2, or 3 days of deacclimation (DEACC1, DEACC2, and DEACC3) at 20/18°C day/night temperatures. Accessions were ordered from the lowest LT_50_ after cold acclimation on the left to the highest on the right. Bars represent means ± standard error (*n* = 3).

To test the activation of ROS-signaling cascades, the transcript levels of the zinc finger transcription factor *ZAT10* and the ferritin complex protein *Fer1* were analyzed. *ZAT10* is a chloroplast ROS-marker gene, which controls extra-plastidic stress tolerance mechanisms, such as activation of ascorbate peroxidase *APx2* (Op den Camp et al., 2003; Mittler et al., 2006; Rossel et al., 2007), while *Fer1* is a superoxide and H_2_O_2_ marker gene involved in iron metabolism (Op den Camp et al., 2003). The *ZAT10* transcript levels (Figure 3A) were slightly increased in all accessions, except Sah-0, after 2 weeks at 4°C, consistent with the regulation observed by Barah et al. (2013). After shifting the plants back to 20°C, the transcript levels in most accessions decreased first and then increased again demonstrating first re-adjustment and then response to secondary ROS-production. No correlation with freezing tolerance was observed.

*Fer1* transcript levels increased only in some of the analyzed accessions after cold acclimation, e.g., N13, WS, Van-0, Col-0, and Can-0 (Figure 3B). The transcript levels decreased quickly in most accessions in the deacclimation phase and transiently re-increased to NA levels at DEACC2. No trend along the LT_50_ profile was observed for *Fer1* prior to and after acclimation. However, N14, N13 and Ms, and Kas-1 showed a similar “down-up-down”-pattern during the first 3 days of the post-cold period.

### Ascorbate levels and redox state

**Figure 4:**
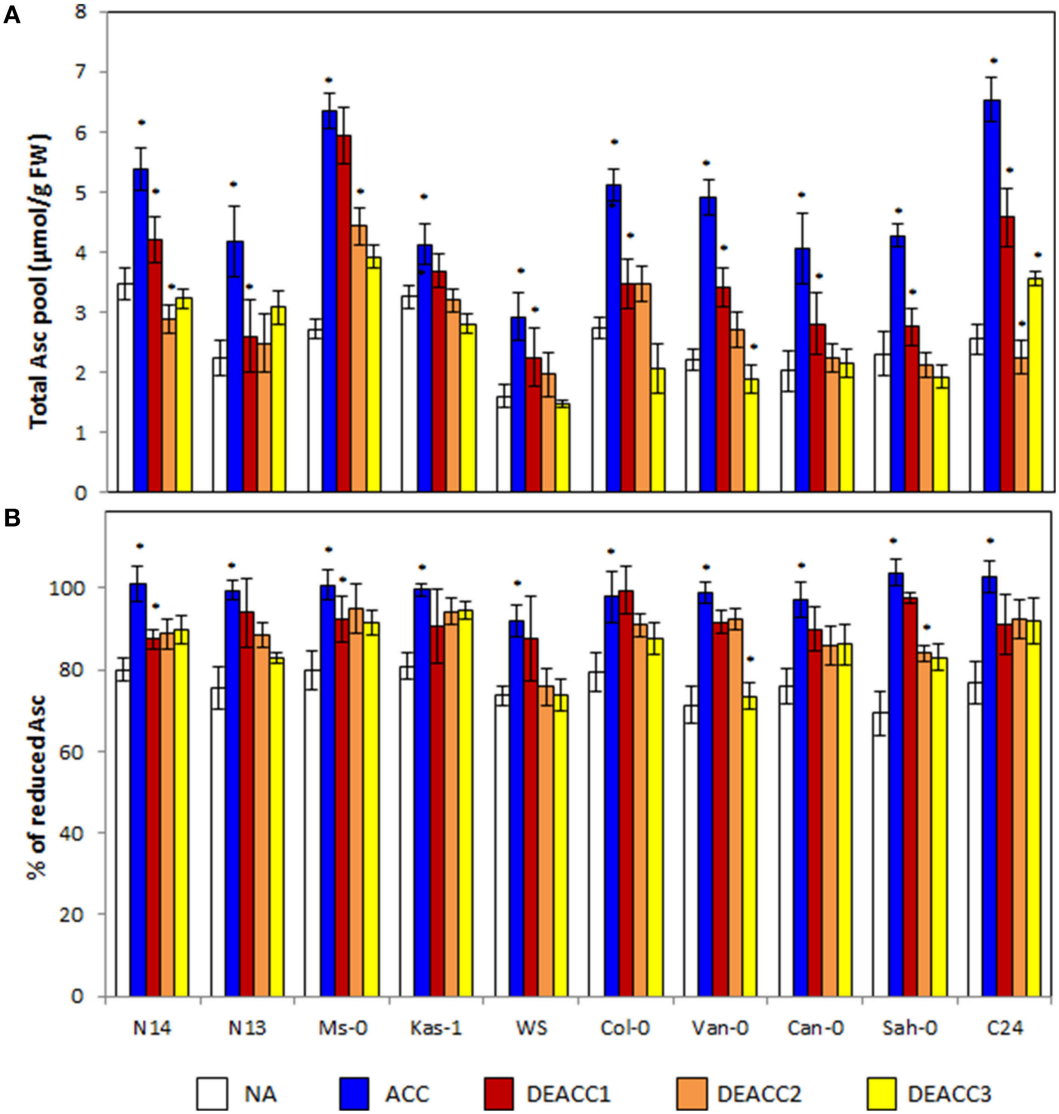
**Ascorbate content (A), and the redox state of the ascorbate pool (B) in the rosettes of the 10 investigated Arabidopsis accessions**. Plants were harvested before (NA) or after 14 days of cold acclimation at 4°C (ACC) and after 1, 2, or 3 days of deacclimation (DEACC1, DEACC2, and DEACC3) at 20/18°C day/night temperatures. Accessions were ordered from the lowest LT_50_ after cold acclimation on the left to the highest on the right. Bars represent means ± standard deviation (*n* = 5). Statistically significant changes (Tukey *post-hoc*, Student *T*-Test; *P* < 0.05) relative to the previous day are labeled with an asterisk.

Ascorbate (Asc) is the major aqueous soluble low molecular weight antioxidant in plants. The ascorbate levels increased during cold acclimation (Figure 4A). The strongest induction was observed in C24 (2.6-fold), Ms-0 (2.3-fold), and Van-0 (2.2-fold), the weakest in Kas-1 (1.3-fold). The ascorbate levels quickly declined in the post-cold phase (Figure 4A). N14, Kas-1, Col-0, Van-0, Can-0, and Sah-0 reached the NA levels within 3 days at 20°C. In N13, Ms-0, and C24, the ascorbate levels were higher after 3 days of deacclimation and in Col-0 lower than before the stress. No trend was observed along the LT_50_ axis.

The ascorbate pool was around 80% reduced prior to the cold-treatment (Figure 4B). In the cold, the ascorbate reduction state (Asc% red) increased in parallel to the ascorbate level. It declined again during deacclimation, but the effect was less than on the ascorbate pool size. After 3 days of deacclimation, the reduction state of the ascorbate pool was still higher than in NA plants in the four accessions with the lowest and the three with the highest LT_50_ values, demonstrating correlation with the change in the R^•^/H_2_O_2_ ratio (Figure 2). In Col-0, despite the fast decrease in the ascorbate concentration, the redox state of the ascorbate pool also declined only slowly.

The Spearman correlation coefficient (r_s_) between the level of the low molecular weight antioxidant ascorbate and the reduction state of the ascorbate pool was high prior to (*r*_s_ = 0.845) and 3 days after the cold treatment (*r*_s_ = 0.800; **Figure 7**). In between, 24 h after the end of the cold period, it dropped to 0.209 due to the fast decrease in the ascorbate level, but slower re-adjustment of the ascorbate redox state.

### Regulation of genes encoding chloroplast antioxidant enzymes

All chloroplast antioxidant enzymes are nuclear encoded and post-translationally targeted to chloroplasts (Pitsch et al., 2010), where they form a network system (Asada, 2000). All, except the thylakoid peroxidase PrxQ, are encoded by small gene families and have, besides PrxQ and the 2CPs, also non-chloroplast isoforms. Stronger than the cytosolic isoforms, the chloroplast ones are prone to oxidative inactivation (Baier et al., 2010). The instability requires a constant supply of *de novo* synthesized proteins (Muthuramalingam et al., 2013).

**Figure 5:**
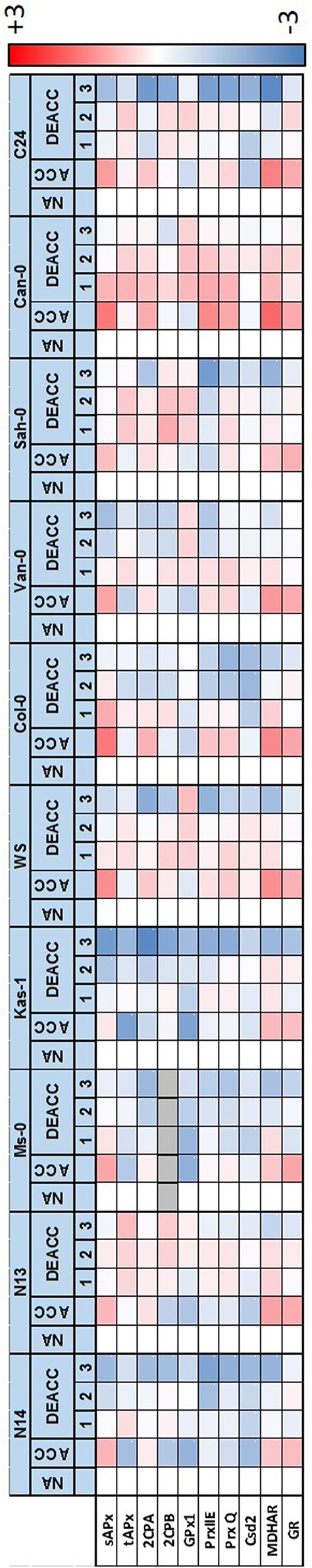
**Heat-map depicting the log_2_-fold change in relative gene expression (Supplementary Figure 1) between non-acclimated (NA), cold-acclimated plants (ACC) and 1–3 day long deacclimated plants (DEACC1–DEACC3) on the scale from −3 (blue) to 3 (red)**. Data below detection level are labeled in gray. Accessions are ordered from the lowest LT_50_ after cold acclimation on the left to the highest on the right.

**Figure 6:**
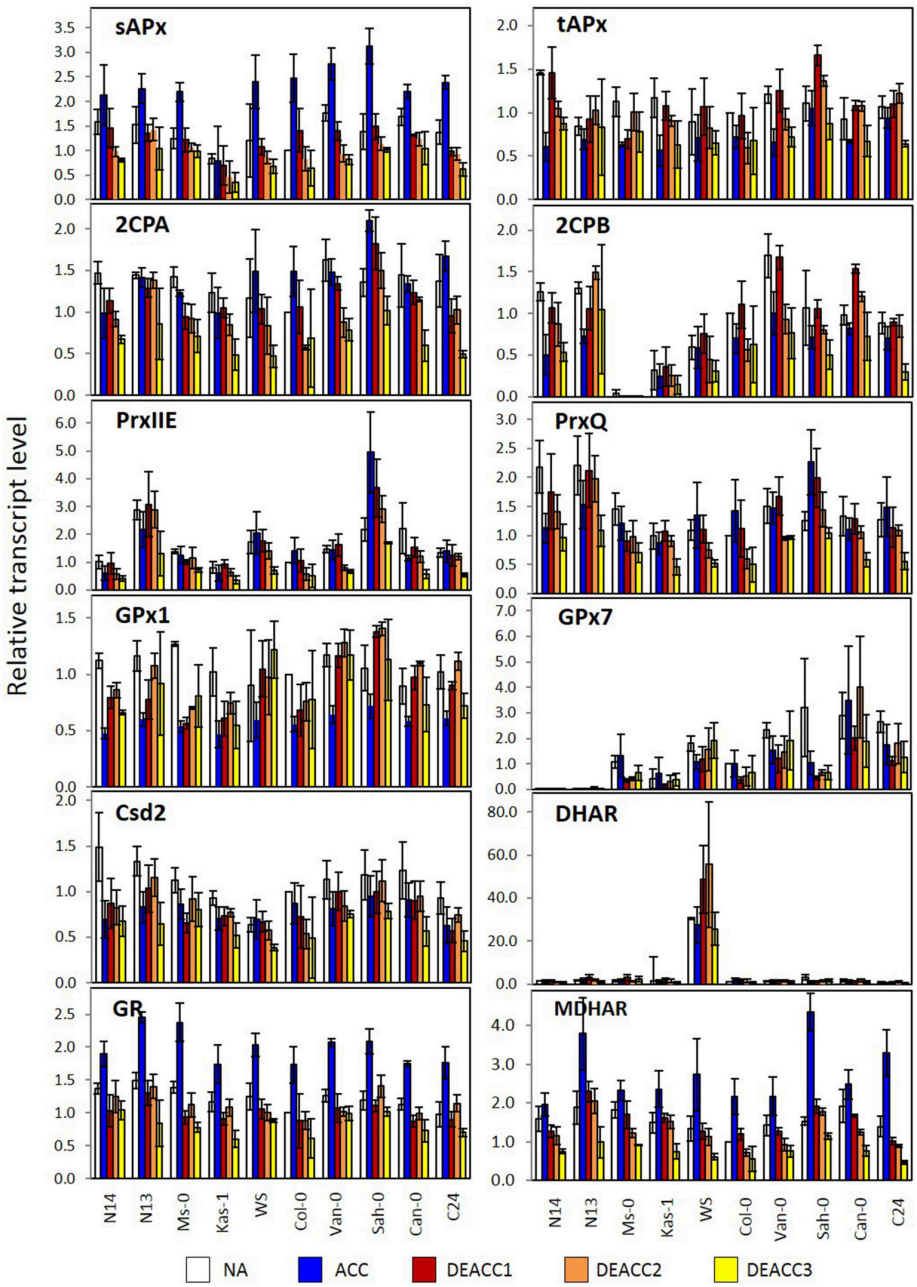
**Transcript levels of genes encoding chloroplast antioxidant enzymes in the rosettes of the 10 investigated Arabidopsis accessions relative to the transcript level in Col-0 prior to the cold treatment**. Plants were harvested before (NA; white) or after (ACC; blue) 14 days of cold acclimation at 4°C and after 1, 2, or 3 days of deacclimation (DEACC1; red, DEACC2; orange and DEACC3; yellow) at 20/18°C day/night temperatures. Accessions were ordered from the lowest LT_50_ after cold acclimation on the left to the highest on the right. Bars represent means ± standard error (*n* = 3).

To study the regeneration capacity, we determined the transcript levels by qRT-PCR with isoform-specific primers (Supplementary Table 2). Special attention was given to the main chloroplast superoxide dismutase *Csd2*, the eight main chloroplast peroxidases (*sAPx, tAPx, 2CPA, 2CPB*, PrxQ, PrxIIE, *GPx1*, and *GPx7*) and to the low molecular weight antioxidant regenerating enzymes *MDHAR, DHAR*, and *GR*. The log_2_ of the transcript levels relative to the NA-levels (Supplementary Figure 1) in the respective accession was compared on a heat map (Figure 5; details enlarged in Supplementary Figure 2A). Because the relative change in the strength of a parameter is often more relevant in signaling and regulation than the absolute level, all transcript regulation data were also further normalized on the transcript level in Col-0 NA plants in each biological replicate to emphasize the relative intensity of regulation (Figure 6): Like all other data in this study, the transcript abundances were arranged along the LT_50_-gradient for acquired freezing tolerance after acclimation (Supplementary Table 1) according to Zuther et al. (2015), to facilitate the comparison with *CBF* and *COR* transcript abundance and metabolite level regulation obtained there.

For *GPx7*, which encodes a weakly expressed peroxiredoxin of the glutathione peroxidase type, an overall gradual trend was observed in NA-plants (Figure 6). The transcript abundance almost constantly increased from hardly detectable levels in N14 and N13 to well detectable levels in accessions with higher LT_50_. The gradient was widely maintained in the cold and during deacclimation.

For *DHAR* more than 10-fold differences in the log_2_ of transcript abundance were observed between WS and the other accessions. The data are depicted in Figure 6, but excluded from Figure 5, since such accession-specific differences, which are several-fold higher than the regulation amplitudes in response to acclimation and deacclimation within the accessions, would have masked the information on the variation in the other accessions. For the same reason, *GPx7*-data are only shown in Figure 6, but not in Figure 5. Most genes for plastid antioxidant enzymes were strongly expressed in the absence of stress with transcript levels close to those of e.g., actin (according to calibrated qRT-PCR data). On top of this high background, changes in the range of 1.2- to 1.5-fold represent strong absolute changes in the PAS.

In response to cold, bidirectional regulation of transcript levels was observed for PAS genes: *sAPx, MDHAR* and *GR* transcript levels were increased in all accessions (Figures 5, 6). As indicated by lighter red color in the heat map (Figure 5; for the acclimation response see also Supplementary Figure 2A), sAPx and MDHAR increased less at 4°C in accessions with more strongly maintained freezing tolerance, e.g., N14, Ms-0, and Kas-1, than in those which lost their freezing tolerance entirely within 24 h (Zuther et al., 2015; Supplementary Table 1). *tAPx, 2CPB, GPx1, PrxIIE, PrxQ*, and *Csd2* transcript levels declined by average stronger in N14, N13, Ms-0, and WS (Figures 5, 6). The mRNAs for the three peroxiredoxins 2CPA, PrxQ, and PrxIIE showed higher accumulation in most of the other accessions.

For statistical evaluation of the significance of the observed trends, we performed cluster analysis of the accessions based on Tukey-HSD variance analysis (*p* < 0.1) of the difference between the transcript levels (normalized to the NA-level in Col-0 as in Figure 6) in acclimated (ACC) and naïve plants (NA) in the three independently cultivated plant sets. In the overall pattern, regulation in N14, N13, Ms-0, and Kas-1 (blue in Supplementary Figure 2B) generally separated from regulation of the accessions with highest LT_50_ (orange in Supplementary Figure 2B). Exceptions from the general pattern are the genes for low molecular weight antioxidant regenerating enzymes *MDHAR* and *GR* as well as *GPx7* and *PrxIIE*. For *MDHAR*, N13 grouped with the high LT_50_ accessions Sah-0 and C24. For *GR*, N13 and Ms-0 formed an independent cluster, which is more similar to the cluster formed by Van-0 and Sah-0 than that formed by N14 and Kas-1, demonstrating accession specific regulation. *GPx7* expression was highly variable throughout the experiment. Due to the high variances in the expression levels between the experiments, no statistically significant clusters could be formed. Similarly, expression regulation of most genes was more variable in Kas-1, Van-0, and Can-0 than in other accessions. No second cluster could be formed for *PrxIIE*, although all other regulation patterns differed significantly from that of N13 and N14. Despite some gene-specific or accession-specific regulation, the analysis confirmed the pattern according to which in N14, N13, Ms-0, and Kas-1 the expression of plastid antioxidant enzymes is by average either significantly less induced or significantly stronger decreased the end of the acclimation period.

After shifting the plants back to 20°C, most transcript levels were inversely regulated on DEACC1 relative to the cold acclimation response (Figure 6). *tAPx* showed the strongest response (Figure 6). Transcript levels increased during the first day of deacclimation and were higher in DEACC1 than in NA plants in Sah-0 and Can-0 and in DEACC3 plants in C24, demonstrating over-compensation of the decrease during the acclimation period.

### Links in the regulation of the PAS genes

**Figure 7:**
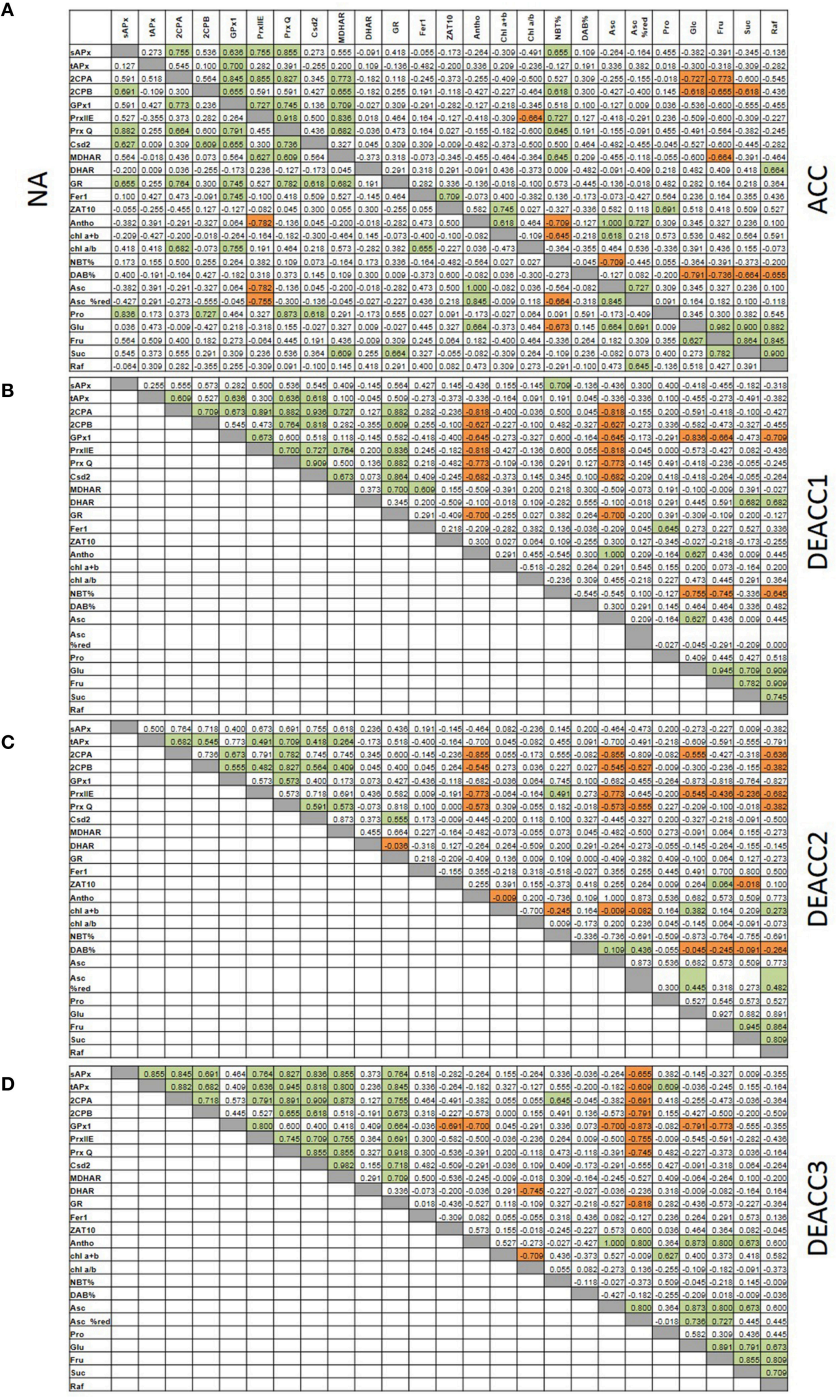
**Spearman correlation matrix with the numerical values of the correlation coefficients (r_*S*_) of all pair-wise correlations for the transcript levels of tested genes and metabolites under non-acclimated conditions (NA; A, left) and after cold acclimation (A, right) and subsequent deacclimation for 1 or 3 days (B–D)**. Significant negative correlations are marked in orange and positive in green. The *p*-values are listed in Supplementary Figure 3.

Spearman correlation coefficients (r_S_; Figure 7; Supplementary Figure 3) were calculated for all data sets obtained in this study and for fructose (Fru), glucose (Glc), sucrose (Suc), and raffinose (Raf) levels determined in Zuther et al. (2015) to analyze them for similarity in regulation. Consistent with the network structure of the chloroplast antioxidant system with redundant, supportive, and successively acting elements (Asada, 2000), the transcript abundance of genes for chloroplast antioxidant enzymes was only weakly linked in NA-plants (Figure 7A). The highest correlation coefficients were observed for the transcript levels of *sAPx, 2CPB, PrxQ, Csd2*, and *GR* (NA; Figure 7). Cold stress adjusted the system: After 2 weeks at 4°C (ACC-plants), correlation was observed for *2CPA, 2CPB, PrxIIE, PrxQ*, and *MDHAR* (Figure 7A). The transcript levels of the two 2-Cys peroxiredoxins were linked to glucose and fructose (Figure 7A). *tAPx, 2CPB, PrxQ*, and *Csd2* formed a partly overlapping second regulatory unit, in which the transcript levels correlated best with the R^•^ levels (NBT%). Correlation with H_2_O_2_-levels was not observed.

After the shift to optimal growth temperatures at the end of the cold period less correlations were observed. *2CPA, 2CPB, GPx1, PrxIIE, PrxQ*, and *Csd2* negatively correlated with the ascorbate levels on DEACC1, while the *sAPx* levels were linked to R^•^ accumulation (NBT%; Figure 7B). On DEACC3, the transcript levels of the peroxidase genes (*APx* and the peroxiredoxins *GPx1, 2CPA, 2CPB, PrxQ*, and *PrxIIE*) were significantly negatively correlated with the redox state of the ascorbate pool (Figure 7D). On the contrary, in the transient post-stress phase on DEACC1 and DEACC2, they were stronger linked to the ascorbate pool size showing an inverse control of enzymatic and non-enzymatic antioxidant protection (Figures 7B,C).

Six weeks old plants are close to bolting. Under our growth conditions, WS, Col-0, Van-0, and C24 had already formed small inflorescences prior to the cold (Supplementary Table 3). Non-flowering Sah-0 and Can-0 showed only little similarity with N14, N13, Ms-0, and Kas-1 in the regulation of PAS genes in the cold (Supplementary Figure 2B) and Sah-0, which still did not bolt at the end of the experiment, resembled strongest bolting C24 in PAS regulation, excluding correlation between the developmental state of the plant material and PAS gene regulation.

### Correlations between the transcript levels of PAS genes and CBF/COR genes

**Figure 8:**
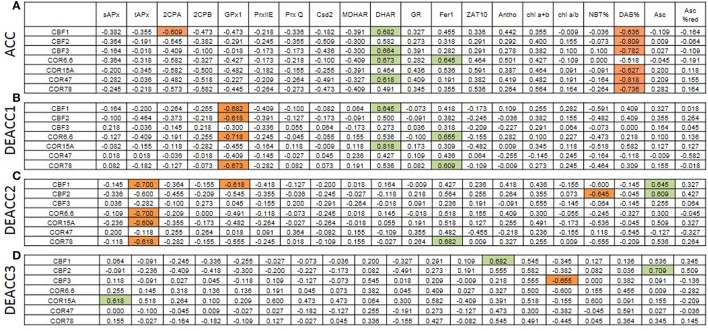
**Spearman correlation matrix with the numerical values of the correlation coefficients (r_*S*_) between the transcript levels of cold-marker genes and all here tested genes and metabolites after cold acclimation (A) and subsequent deacclimation for 1 or 3 days (B–D)**. Significant negative correlations are marked in orange and positive in green. The *p*-values are listed in Supplementary Figure 4.

For analysis of the impact of the regulation of various PAS genes on the regulation on C-repeat binding factors (*CBF*) and their cold-regulated target genes (*COR*), transcript abundance regulation upon acclimation and deacclimation of *CBF*s and *COR* genes (taken from Zuther et al., 2015) were compared with the transcript abundance regulation of PAS genes and metabolic indicators for the chloroplast redox effect (Figure 8; Supplementary Figure 4). After 2 weeks of cold acclimation, the transcript levels of all tested *CBF*s and *COR* genes, except COR6.6, were significantly negatively correlated with H_2_O_2_-levels (DAB%; Figure 8). This result is consistent with the observation that insufficient H_2_O_2_ detoxification slows *CBF1* and *COR* expression (Maruta et al., 2012).

*CBF1, CBF3, COR6*.6, and *COR78* were widely co-regulated with *DHAR* (Figure 8), which promotes ascorbate recycling and, consequently, ascorbate peroxidase function in chloroplasts. *CBF1* was weakly negatively linked to *2CPA* which is a highly abundant alkyl hydroperoxidase and H_2_O_2_ detoxifying chloroplast peroxidase (König et al., 2002). *2CPA* transcripts increased in cold strongest in the accessions with lowest induction of *CBF*- and *COR*-genes (WS-C24) pointing out 2CPA as a driver of H_2_O_2_-controlled *CBF*- and *COR*-gene regulation.

On DEACC1, the links to *2CPA* transcript levels diminished and those to *GPx1*, another chloroplast peroxiredoxin, got stronger (Figure 8). On the next day (DEACC2), the ascorbate level had an increased impact on *CBF*-regulation and the transcript abundance of *CBF* and *COR* genes correlated more negatively with *tAPx* regulation (Figure 8).

## Discussion

Cold deacclimation is a fast process, as compared to cold acclimation (Kalberer et al., 2006). It is assumed to be mainly passive and to require hardly any energy resources (Thomashow, 1999; Browse and Lange, 2004). Recently, we observed that the metabolic pattern is largely, but not completely, re-set after 1 day of deacclimation in those Arabidopsis accessions that had lowest LT_50_ temperatures after cold acclimation (Zuther et al., 2015). In the present study we showed in the same plant material that in the accessions that halted cold deacclimation for days at optimal growth temperature (N14–Kas-1), expression of various PAS genes is lower after cold acclimation than in the other accessions. The transcript levels for the ROS-detoxifying PAS enzymes were in general either more strongly decreased or were less activated in the accessions with low LT_50_ (Figures 5, 6; Supplementary Figure 2). For the genes encoding the low-molecular weight regenerating enzymes MDHAR and GR the same regulation trend was observed in N14, Ms-0, and Kas-1 or N14 and Kas-1, respectively, indicating a wider general effect of lower expression of PAS genes in accessions with low LT_50_, but accession specific exceptions. The ROS levels did not differ significantly between the accessions after acclimation (Figure 2), indicating that the low PAS capacity was compensated e.g., by cold-induction of the extra-plastidic antioxidant system (EAS; Distelbarth et al., 2013; Chen et al., 2014). Downregulation of PAS and up-regulation of EAS shift the ROS detoxification potentials within the cell and can impact on ROS detoxification upon the shift from cold to optimal growth conditions. A higher risk for Mehler-reaction activity (Mehler, 1951) strains the PAS upon the on-set of the deacclimation phase. Consistently, R^•^ accumulated in the deacclimation phase (Figure 2A), while the H_2_O_2_ levels decreased (Figure 2B) by the action of cold-induced extra-plastidic peroxidases and catalase (O'Kane et al., 1996; Du et al., 2008).

### Accession-specific Csd2 regulation

In the comparison of the Arabidopsis accessions, R^•^ levels increased stronger in most accessions with low LT_50_. In the same lines, the decrease in the H_2_O_2_ levels was delayed, demonstrating that the post-cold-stress correlates with photooxidative ROS formation and delayed superoxide detoxification. In chloroplasts, Csd2 is the main chloroplast superoxide dismutase in Arabidopsis and essential during photooxidative stress (Kliebenstein et al., 1998; Yabuta et al., 2002; Sales et al., 2013). Here, *Csd2* transcript levels remained low in all accessions during deacclimation (Figure 6), although the gene is strongly stress-inducible (Kliebenstein et al., 1998; Xing et al., 2013), mainly transcriptionally controlled in response to temperature changes (Juszczak and Baier, 2012) and responds gradually to temperature variation (Juszczak and Baier, 2012). That *Csd2* transcript levels were not restored in any accession during deacclimation (Figures 5, 6), demonstrates down-regulation depending on the previous acclimation process. The accessions with the lowest LT_50_ after cold acclimation showed the largest decrease in *Csd2* transcript abundance in the cold and de-regulation after deacclimation was strongest in N14 and N13 (Figures 5, 6; Supplementary Figure 2). Escaping O${}_{2}^{-}$ by transiently insufficient Csd2 activity results in a severe reduction of photosynthetic activity and plant growth retardation and promotes O${}_{2}^{-}$ signaling (Bowler et al., 1994; Ogawa et al., 1997; Xing et al., 2013). Accumulation of R^•^ during the first days of deacclimation (Figure 2), demonstrated that low *Csd2* expression was also not compensated by other antioxidant enzymes.

### The CBF regulon under PAS control

Microarray analysis of *Csd2*-knockdown plants gives no indication for regulation of the *CBF* genes and their downstream genes by O${}_{2}^{-}$ (Rizhsky et al., 2003). *CBF1* expression and the CBF-regulon are suppressed by chloroplast H_2_O_2_ in *tAPx*-silenced plants (Maruta et al., 2012) and by insufficient extra-plastidic H_2_O_2_ detoxification in catalase-knockdown lines (Vanderauwera et al., 2005) at ambient temperature, suggesting regulation of this signal transduction pathway by H_2_O_2_ of chloroplast and of extra-plastidic origin. O${}_{2}^{-}$ and H_2_O_2_ drive distinct signal transduction cascades (Gadjev et al., 2006). Maintenance of *CBF1*-expression slightly above the levels prior to cold was only observed in N14, N13, Ms-0, and Kas-1 (Zuther et al., 2015). Prolonged *CBF1* activation was accompanied by weaker expression of the genes for chloroplast peroxidases and *CSD2* at the end of the cold-period (Figures 5, 6; Supplementary Figure 2) and a delayed onset of the shift in the R^•^/H_2_O_2_ ratio during deacclimation (delayed to DEACC2; Figure 2).

*tAPx* transcript levels correlated negatively with *CBF1* transcript levels and with transcript levels of several COR genes on DEACC2 (Figure 8), suggesting that the regulatory circuitry on *CBF*-regulation postulated by Maruta et al. (2012) either does not apply in the post-cold deacclimation regulation or, as already discussed by Maruta et al. (2012), is not specific to *tAPx*. Our data support the latter assumption: In our experiment, most genes encoding PAS enzymes were on the average either less induced or more decreased at the end of the cold period in the accessions which incompletely switch off their cold-acclimation responses during deacclimation (N14–Kas-1; Zuther et al., 2015; Figures 5, 6; Supplementary Figure 2). Correlation analysis on 10 accessions after cold acclimation and during de-acclimation indicates that the halted expression of various cold-marker genes depends on the post-cold capacity and enzyme composition of the PAS (Figure 7). Already one of the first publications on transgenic Arabidopsis with modified expression levels of chloroplast antioxidant enzymes (Allen et al., 1997) showed that the regulation of stress protection depends on a delicate balance of the antioxidant protection mechanisms. In N14, N13, Ms-0, and Kas-1 the PAS transcript composition was shifted relative to the NA-status (Figures 5, 6; Supplementary Figure 2). This regulation might weaken the chloroplast-intrinsic protection against photo-oxidative stress upon variation in the environmental conditions, such as the transfer of cold and lower light intensity acclimated plants to optimal growth conditions.

In the series of 10 Arabidopsis accessions (Figure 6; Supplementary Figure 2) strongest differences between N14, N13, Ms-0, Kas-1 (accessions with low LT_50_ after cold acclimation), and the other accessions in ACC plants were observed for *2CPA* and *sAPx*. *2CPA* is the most abundant chloroplast peroxidase, *sAPx* the one with the highest catalytic activity (König et al., 2002; Dietz et al., 2006). At optimal temperature, these two genes are regulated by feed-back loops according to which low activity of one enzyme activates expression of the other gene (Baier et al., 2000; Kangasjärvi et al., 2008; Pulido et al., 2010). In the cold, weaker expression of both genes demonstrates loss of the feed-back effects, which are essential to stabilize the overall plastid peroxidase activity. In response, plastid ROS may activate ROS signaling cascades stronger (Gechev et al., 2002; Rossel et al., 2007). Induction of extra-plastidic protection, such as induction of catalase and peroxidases, during cold periods has been well described in literature for many plants, including Arabidopsis (O'Kane et al., 1996; Du et al., 2008). Cold-acclimation widely attenuated the overall H_2_O_2_ levels in all accessions (Figure 2).

**Figure 9:**
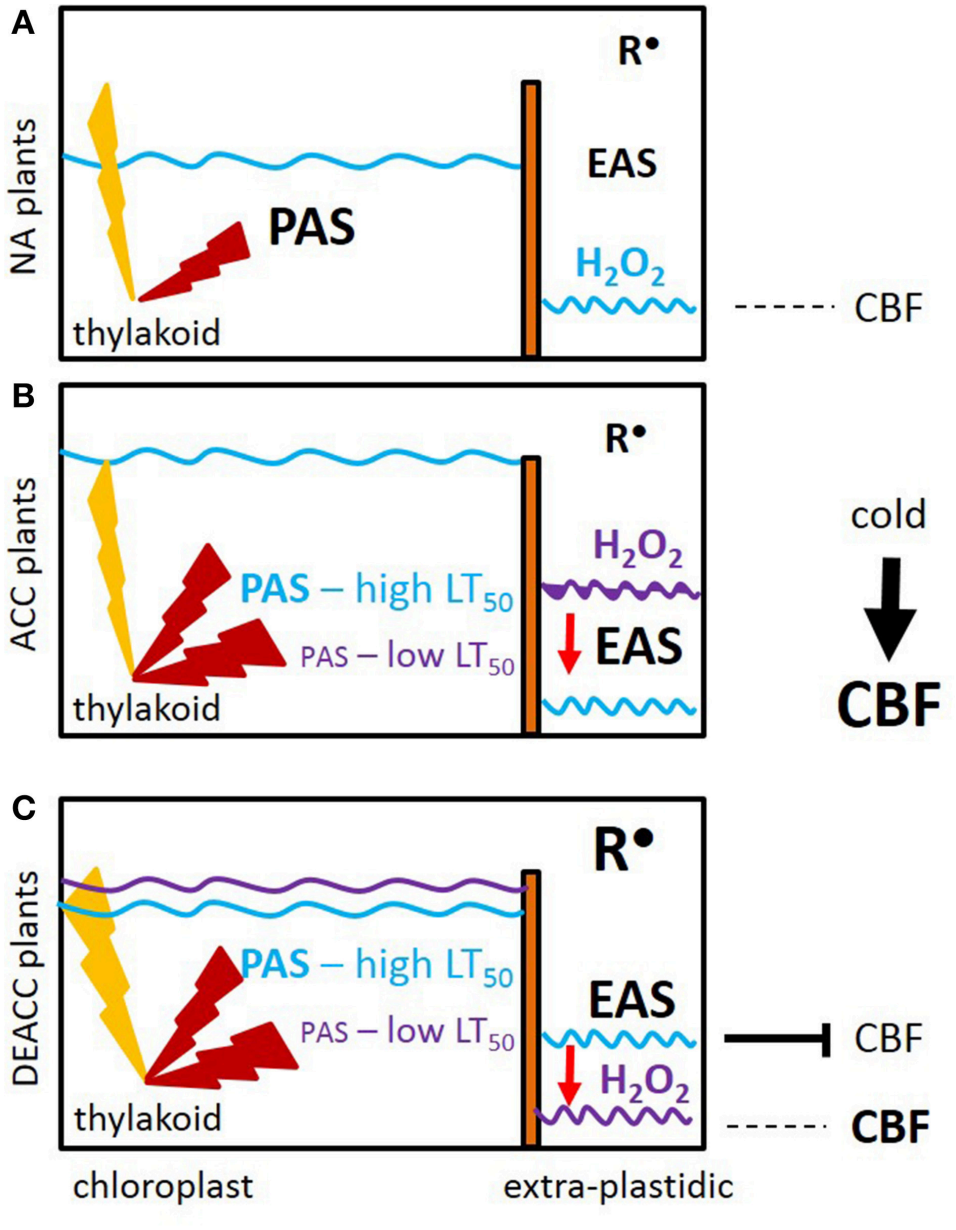
**Prolonged activation of CBF-controlled cold-responses (CBF) as a consequence of decreased activity of the plastid antioxidant system (PAS)**. **(A)** Prior to cold (NA plants), the plastid, and extra-plastidic antioxidant systems (PAS and EAS) are balanced. Light (yellow flash) induced ROS (red flash) are widely antagonized by the PAS activities and only a small amount of ROS pass the PAS. **(B)** In the acclimated state (ACC plants), the PAS capacity is stronger decreased in the accessions with low LT_50_. The decrease in chloroplast peroxidase activities strongest correlates with post-cold CBF regulation. Attenuation by EAS induction antagonizes (red arrow) accumulation of H_2_O_2_ (purple → blue waved line). **(C)** Upon the on-set of deacclimation (DEACC), cold-induction of CBF stops. The inactivation is slowed by the shift (purple line compared to blue line) in H_2_O_2_ detoxification in the lines with lower LT_50_ due to cold-dependent setting of the antioxidant capacities inside chloroplasts (PAS) and in other cellular compartments (EAS). Radicals (R^•^) accumulate in parallel. Correlation analysis links H_2_O_2_ detoxification, but not R^•^, to *CBF*-regulation.

Our data let us to the hypothesis depicted in Figure 9: The EAS effect is shown by shifting the H_2_O_2_ levels from higher (purple line) to lower levels (blue line; Figure 9B). When the accessions with weaker PAS and stronger EAS activities (ACC plants) were shifted back to normal growth temperatures and light intensities, the PAS got more strained and was (transiently) overwhelmed e.g., by photooxidative ROS production, as noticeable by the delay in the decrease in the H_2_O_2_ levels from DEACC1 to DEACC2 (Figure 2–accessions with low LT_50_). The high EAS activity, as acquired during the cold period (O'Kane et al., 1996; Du et al., 2008), can be expected to have counteracted H_2_O_2_ accumulation in the cytosol. Compared to accessions with less inactivation of the PAS and, consequently, weaker compensation of the EAS in the cold (the high LT_50_ accessions), the shift in the PAS-EAS-activities keeps the extra-plastidic H_2_O_2_ pool lower in the accessions with low LT_50_, while the plastidic H_2_O_2_ pool might be elevated due to insufficient PAS activity. In response, CBF-expression is less inhibited and enables prolonged CBF-expression after the transfer to optimal growth conditions (Figure 9C).

In summary, we conclude that the driving forces for prolonged activation of cold-acclimation responses in N14, N13, Ms-0, and Kas-1 were established already in the cold by weaker expression of PAS genes and secondary activation of EAS. The accessions originate from cold-continental habitats in Russia and the Kashmir mountains, where the plants face late springs and short vegetation periods. In these areas, stronger inactivation of the PAS may be a strategy to keep cold-acclimation reactions partly activated and ease their re-activation by future cold stresses for some days.

## Author contributions

IJ and EZ performed the experiments and qRT-PCR analysis, IJ determined the pigment and ROS-levels, JC did the ascorbate analysis. IJ and JC drafted parts of the manuscript. EZ, DH, and MB supervised the project and finalized the manuscript.

## Funding

German Research foundation (CRC 973—Priming and Memory of Organismic Responses to stress—projects A3 and C4) and the FU-Berlin.

### Conflict of interest statement

The authors declare that the research was conducted in the absence of any commercial or financial relationships that could be construed as a potential conflict of interest.

## Supplementary material

Supplementary Table 1 **List of the means for LT_50_ values obtained in the study by Zuther et al. (2015) in the 10 accession prior to the cold treatment (NA), after cold acclimation (ACC) and 1 and 3 days after re-transfer of the plants to optimal growth conditions**. Freshly harvested leaves were frozen to various temperatures between −1 and −25°C. The LT_50_ was determined based on electrolyte leakage.

Supplementary Table 2 **List of primers used in this study**. The primer pair marked with an asterisk was used for amplification of *sAPx* only in Kas-1.

Supplementary Table 3 **Developmental effect on bolting**. Range of inflorescence lengths of the 10 accession prior to the cold treatment (NA), after cold acclimation (ACC) and 2 days after re-transfer of the plants to optimal growth conditions (DEACC2).

Supplementary Figure 1 **Relative expression (2^−ΔCt^) of all genes indicated in Figures 5, 6**. Transcript abundances were determined by qRT-PCR and normalized on the transcript abundance of four reference genes. Relative transcript abundance of all genes normalized on the transcript levels in Col-0 prior to the cold treatment. The data represent the means from three independent experiments, each with five plants per treatment.

Supplementary Figure 2 **(A)** Heat-map depicting the log_2_-fold change in relative gene expression between non-acclimated and cold-acclimated plants on the scale from −3 (blue) to +3 (red) as extracted from Figure 5 for better comparison. Accessions are ordered from the lowest LT_50_ after cold acclimation on the left to the highest on the right. **(B)** Cluster and significance analysis of transcript level changes during acclimation (ACC–NA) in the 10 Arabidopsis accessions based on Tukey-HSD test (*p* < 0.1). The numbers give the means of the difference of ACC and NA transcript values after normalization of the data on the respective NA-level in Col-0 (as in Figure 6). Statistically significant changes (Tukey-LSD, *p* < 0.1, *n* = 3) are written with straight numbers, non-significant changes in italics. The “minus” in front to the numbers demonstrates that transcript level decreased in the accession during cold acclimations. The various colors stand for different significance groups according to the Tukey-HSD test (*p* < 0.1) on similarities and differences of the regulation pattern of the respective gene in the 10 Arabidopsis accessions. In each lane, the prominent cluster for low LT_50_ accessions is marked blue and the prominent cluster for the high LT_50_ accessions is marked orange.

Supplementary Figure 3 ***p*-values of the correlation coefficients of all pair-wise correlations for the transcript levels of tested genes and metabolites under non-acclimated conditions (NA; A, left) and after cold acclimation (A, right) and subsequent deacclimation for 1 or 3 days (B–D)**. *p* < 0.001 are labeled in red, < 0.01 in orange and < 0.05 in yellow.

Supplementary Figure 4 ***p*-values of the correlation coefficients of all pair-wise correlations for the transcript levels of tested genes and metabolites under non-acclimated conditions (NA; A, left) and after cold acclimation (A, right) and subsequent deacclimation for 1 or 3 days (B–D)**. *p* < 0.001 are labeled in red, < 0.01 in orange and < 0.05 in yellow.

Supplementary Figure 5 **Data depicted in Figure 1 normalizes for each accession on the levels of NA plants, Chlorophyll contents (A), Chl a/b ratio (B), and anthocyanin contents (C)**. Bars represent means ± standard deviation (*n* = 9).

## Abbreviations

2CPA/2CPB: 2-Cys peroxiredoxin A/B
ACC: plants acclimated to 4°C
Antho: anthocyanins
Asc: ascorbate
Asc % red: percentage of reduced ascorbate
CBF: C-repeat-binding factor
Chl: chlorophyll
Chl a+b: total chlorophyll
Chl a/b: chlorophyll a/b ratio
COR: cold-regulated
Csd2: Cu/Zn-superoxide dismutase 2
DAB: 3,3′-diaminobenzidine
DAB%: % of DAB stained leaf area
DEACC: deacclimation
DHAR: dehydroascorbate reductase
EAS: extra-plastidic antioxidant system
Fru: fructose
Glc: glucose
GPx: glutathione reductase
LT_50_: temperature at which 50% damage occurred
MDHAR: monodehydroascorbate reductase
NA: non-acclimated plants
NBT: nitroblue tetrazolium
NBT%: % of NBT stained leaf area
PAS: plastid antioxidant system
Pro: proline
ROS: reactive oxygen species
sAPx: stromal ascorbate peroxidase
R^•^: radical
Raf: raffinose
SD: standard deviation
SEM: standard error
SOD: superoxide dismutase
Suc: sucrose
tAPx: thylakoid-bound ascorbate peroxidase.
