# Supplementary material for: Natural Variation of Cold Deacclimation Correlates with Variation of Cold-Acclimation of the Plastid Antioxidant System in Arabidopsis thaliana Accessions
Source: Front Plant Sci. 2016 Mar 17;7:305. doi: 10.3389/fpls.2016.00305 (PMC4794505; doi:10.3389/fpls.2016.00305)
Supplement: Supplementary Table 1 — List of the means for LT50 values obtained in the study by Zuther et al. (2015) in the 10 accession prior to the cold treatment (NA), after cold acclimation (ACC) and 1 and 3 days after re-transfer of the plants to optimal growth conditions. Freshly harvested leaves were frozen to various temperatures between −1 and −25°C. The LT50 was determined based on electrolyte leakage. [file Table1.docx]

**Suppl. Tab.1**

| **accession** | **LT_50_ NA (°C)** | **LT_50_**  **ACC**  **(°C)** | **LT_50_ DEACC3 (°C)** |
| --- | --- | --- | --- |
| **N14** | -7.4 | -12.0 | -8.9 |
| **N13** | -5.5 | -11.9 | -9.3 |
| **Ms-0** | -7.7 | -11.9 | -8.5 |
| **Kas-1** | -4.7 | -11.9 | -8.9 |
| **WS** | -6.0 | -10.4 | -8.4 |
| **Col-0** | -5.3 | -9.1 | -7.7 |
| **Van-0** | -6.0 | -8.8 | -7.4 |
| **Sah-0** | -4.7 | -6.2 | -6.1 |
| **Can-0** | -4.3 | -5.9 | -5.8 |
| **C24** | -4.6 | -5.3 | -5.6 |
