# Supplementary material for: Natural Variation of Cold Deacclimation Correlates with Variation of Cold-Acclimation of the Plastid Antioxidant System in Arabidopsis thaliana Accessions
Source: Front Plant Sci. 2016 Mar 17;7:305. doi: 10.3389/fpls.2016.00305 (PMC4794505; doi:10.3389/fpls.2016.00305)
Supplement: Supplementary Table 2 — List of primers used in this study. The primer pair marked with an asterisk was used for amplification of sAPx only in Kas-1. [file Table2.docx]

**Suppl. Tab.2**

| **Gene** | **Gene code** | **Forward and reverse primer** |
| --- | --- | --- |
| ***Csd2*** | At2g28190 | Fwd.: ATGACACACGGAGCTCCAGAA  Rev: ATTGTTGTTTCTGCCACGCCA |
| ***sAPx*** | At4g08390 | Fwd: AGAATGGGATTAGATGACAAGGAC  Rev: TCCTTCTTTCGTGTACTTCGT |
| ***sAPx^*^*** | At4g08390 | Fwd: CTACTGCTATAGAGGAAGCTGG  Rev: TGGAAGCCTTCCTTCTTCTG |
| ***tAPx*** | At1g77490 | Fwd: GCTAGTGCCACAGCAATAGAGGAG  Rev: TGATCAGCTGGTGAAGGAGGTC |
| ***GPx1*** | At2g25080 | Fwd: TCCCTGCAATCAGTTTGGTTTCC  Rev: TGGTCCATTCACGTCAACCTTATC |
| ***GPx7*** | At4g31870 | Fwd: CGTTAACGTTGCGTCAAGATGTGG  Rev: TGACCTCCAAATTGATTGCAAGGG |
| ***2CPA*** | At3g11630 | Fwd: CCCAACAGAGATTACTGCCT  Rev: ATAGTTCAGATCACCAAGCCC |
| ***2CPB*** | At5g06290 | Fwd: TCATACCCTCTTCCTCGGCATC  Rev: ACCGACCAGTGGTAAATCATCAGC |
| ***PrxIIE*** | At3g52960 | Fwd: CAGTAACCGTCTCATCCCTAAC  Rev: TGAGTTCTCCGGCTTTGGATACG |
| ***PrxQ*** | At3g26060 | Fwd: AGATGACTCTGCTTCTCACAAGGC  Rev: TCCCTGGCAATGCTCCAAACAG |
| ***MDHAR*** | At1g63940 | Fwd: TGGGAGAAACAGTGGAGGTTGG  Rev: TGGTAGAAGCTGGAACTCCTCAG |
| ***DHAR*** | At1g19550 | Fwd: CTCGAGGAGAAGTATCCTGATC  Rev: CCATCGTGACTCTTGAGATGGTTC |
| ***GR*** | At3g54660 | Fwd: GAAATTCCGCAAAGACTCCTC  Rev: CAGACACAATGTTCTCCTTATCAG |
| ***Act2*** | At3g18780 | Fwd: AATCACAGCACTTGCACCAAGC  Rev: CCTTGGAGATCCACATCTGCTG |
| ***GAPDH*** | At1g13440 | Fwd: TTGGTGACAACAGGTCAAGCA  Rev: AAACTTGTCGCTCAATGCAATC |
| ***EXPRS*** | At2g32170 | Fwd: ATCGAGCTAAGTTTGGAGGATGTAA  Rev: TCTCGATCACAAACCCAAAATG |
| ***PDF2*** | At1g13320 | Fwd: TAACGTGGCCAAAATGATGC  Rev: GTTCTCCACAACCGCTTGGT |
