# Supplementary material for: Natural Variation of Cold Deacclimation Correlates with Variation of Cold-Acclimation of the Plastid Antioxidant System in Arabidopsis thaliana Accessions
Source: Front Plant Sci. 2016 Mar 17;7:305. doi: 10.3389/fpls.2016.00305 (PMC4794505; doi:10.3389/fpls.2016.00305)
Supplement: Supplementary Table 3 — Developmental effect on bolting. Range of inflorescence lengths of the 10 accession prior to the cold treatment (NA), after cold acclimation (ACC) and 2 days after re-transfer of the plants to optimal growth conditions (DEACC2). [file Table3.docx]

**Suppl. Tab.3**

| **accession** | **IF length**  **NA**  **(cm)** | **IF length ACC**  **(cm)** | **IF length DEACC2 (cm)** |
| --- | --- | --- | --- |
| **N14** | 0 | 0 | 0 |
| **N13** | 0 | 0 | 0 |
| **Ms-0** | 0 | 0 | 0 |
| **Kas-1** | 0 | 0 | 0 |
| **WS** | 3-8 | 10-18 | 18-24 |
| **Col-0** | 0.5-2 | 2-6 | 4-14 |
| **Van-0** | 0.2-0.5 | 2-3 | 2-7 |
| **Sah-0** | 0 | 0 | 0 |
| **Can-0** | 0 | 0 | 0 |
| **C24** | 0-0.2 | 2-5 | 2-14 |
