# Supplementary figures and images for: Natural Variation of Cold Deacclimation Correlates with Variation of Cold-Acclimation of the Plastid Antioxidant System in Arabidopsis thaliana Accessions
Source: Front Plant Sci. 2016 Mar 17;7:305. doi: 10.3389/fpls.2016.00305 (PMC4794505; doi:10.3389/fpls.2016.00305)

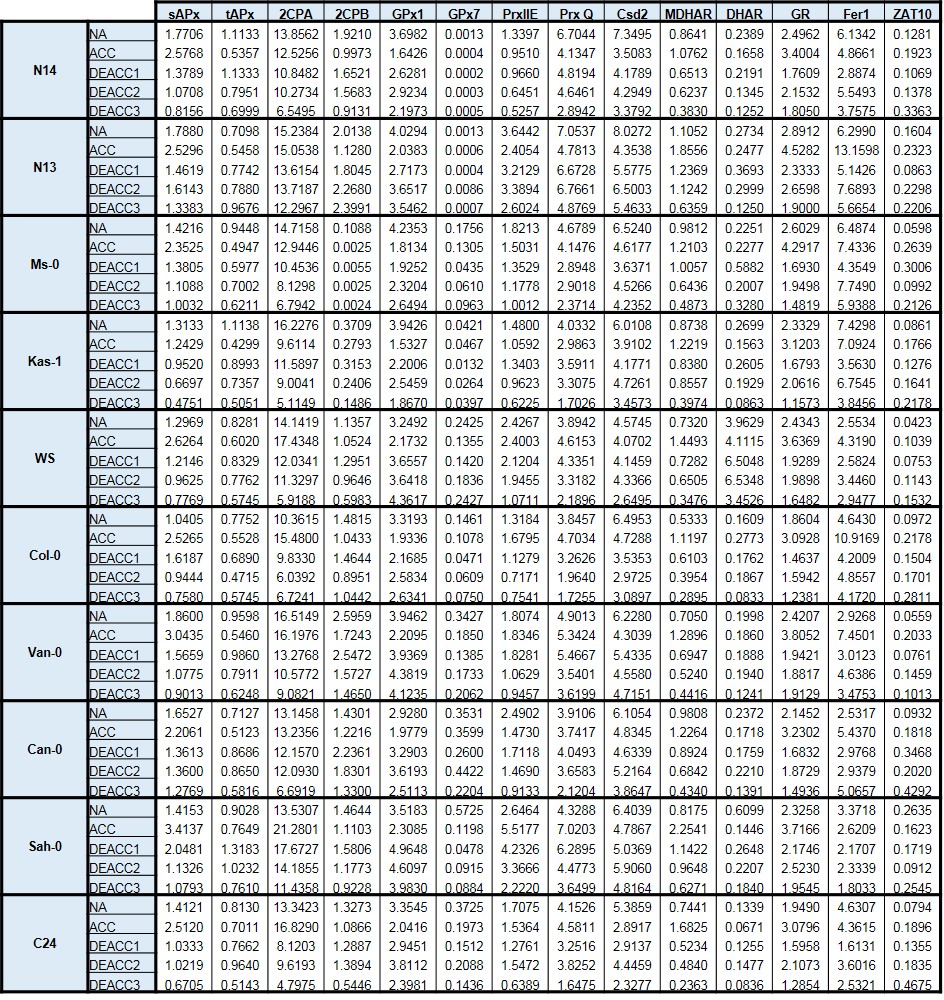

Supplement: Supplementary Figure 1 — Relative expression (2−ΔCt) of all genes indicated in Figures 5, 6. Transcript abundances were determined by qRT-PCR and normalized on the transcript abundance of four reference genes. Relative transcript abundance of all genes normalized on the transcript levels in Col-0 prior to the cold treatment. The data represent the means from three independent experiments, each with five plants per treatment. [file Image1.JPEG]

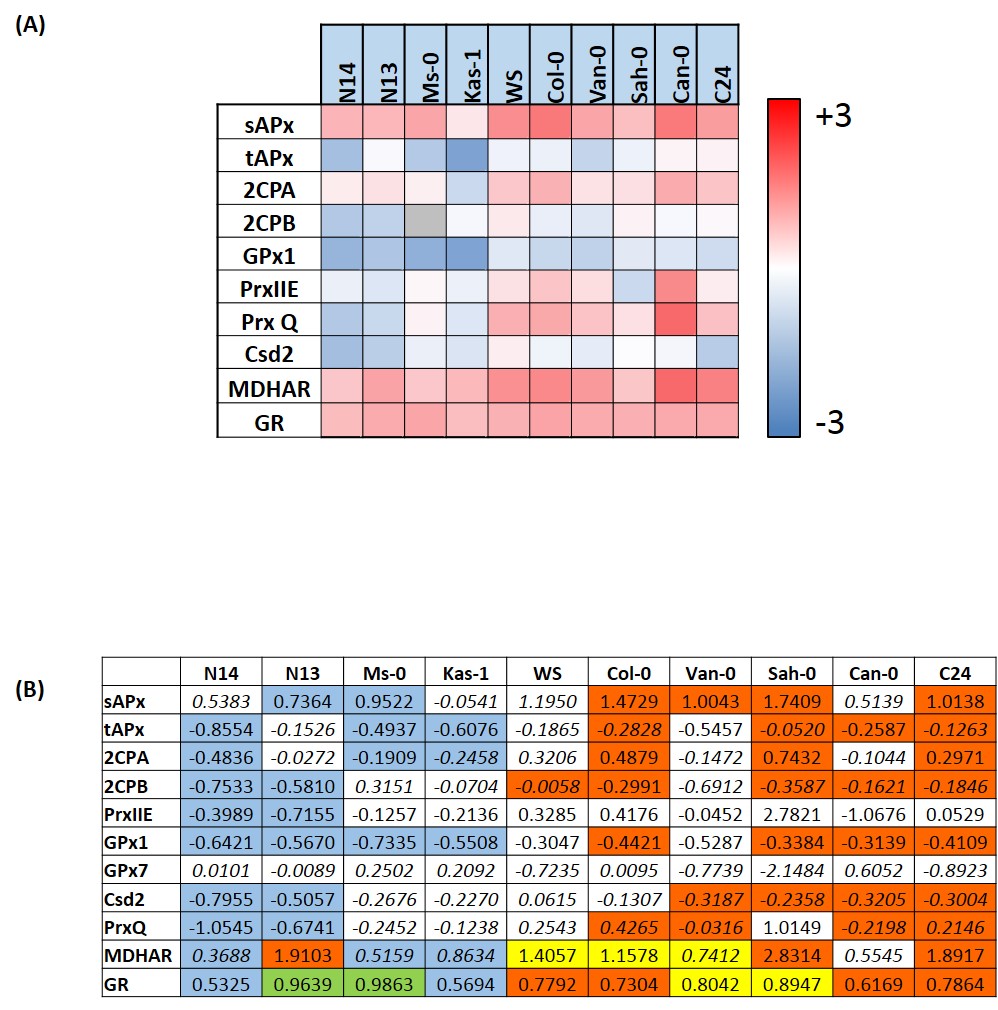

Supplement: Supplementary Figure 2 — (A) Heat-map depicting the log2-fold change in relative gene expression between non-acclimated and cold-acclimated plants on the scale from −3 (blue) to +3 (red) as extracted from Figure 5 for better comparison. Accessions are ordered from the lowest LT50 after cold acclimation on the left to the highest on the right. (B) Cluster and significance analysis of transcript level changes during acclimation (ACC–NA) in the 10 Arabidopsis accessions based on Tukey-HSD test (p < 0.1). The numbers give the means of the difference of ACC and NA transcript values after normalization of the data on the respective NA-level in Col-0 (as in Figure 6). Statistically significant changes (Tukey-LSD, p < 0.1, n = 3) are written with straight numbers, non-significant changes in italics. The “minus” in front to the numbers demonstrates that transcript level decreased in the accession during cold acclimations. The various colors stand for different significance groups according to the Tukey-HSD test (p < 0.1) on similarities and differences of the regulation pattern of the respective gene in the 10 Arabidopsis accessions. In each lane, the prominent cluster for low LT50 accessions is marked blue and the prominent cluster for the high LT50 accessions is marked orange. [file Image2.JPEG]

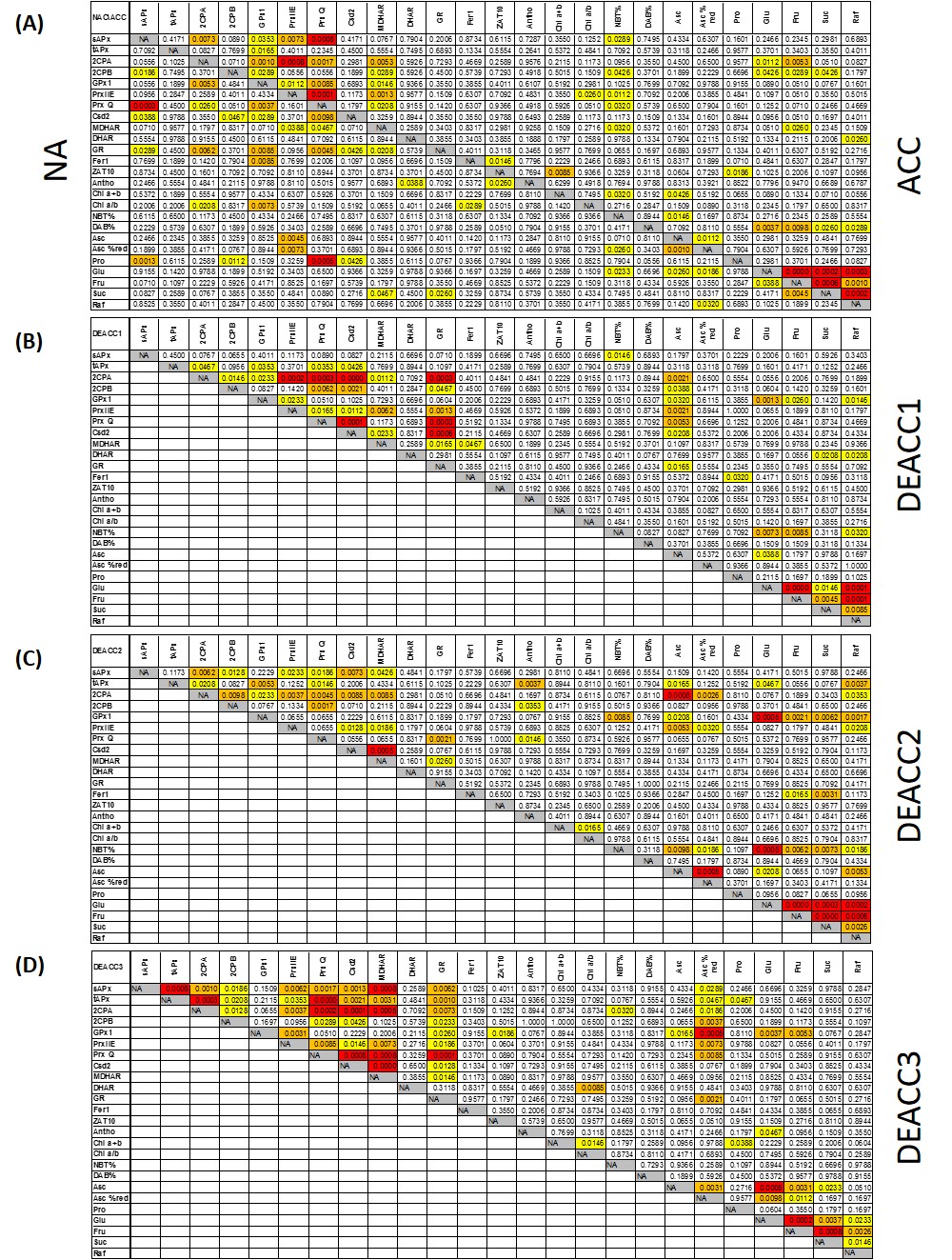

Supplement: Supplementary Figure 3 — p-values of the correlation coefficients of all pair-wise correlations for the transcript levels of tested genes and metabolites under non-acclimated conditions (NA; A, left) and after cold acclimation (A, right) and subsequent deacclimation for 1 or 3 days (B–D). p < 0.001 are labeled in red, < 0.01 in orange and < 0.05 in yellow. [file Image3.JPEG]

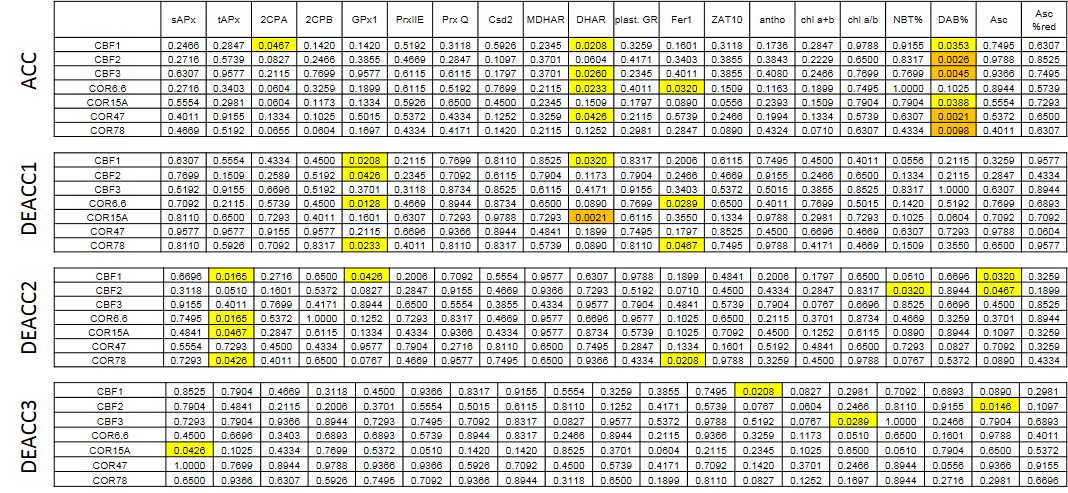

Supplement: Supplementary Figure 4 — p-values of the correlation coefficients of all pair-wise correlations for the transcript levels of tested genes and metabolites under non-acclimated conditions (NA; A, left) and after cold acclimation (A, right) and subsequent deacclimation for 1 or 3 days (B–D). p < 0.001 are labeled in red, < 0.01 in orange and < 0.05 in yellow. [file Image4.JPEG]

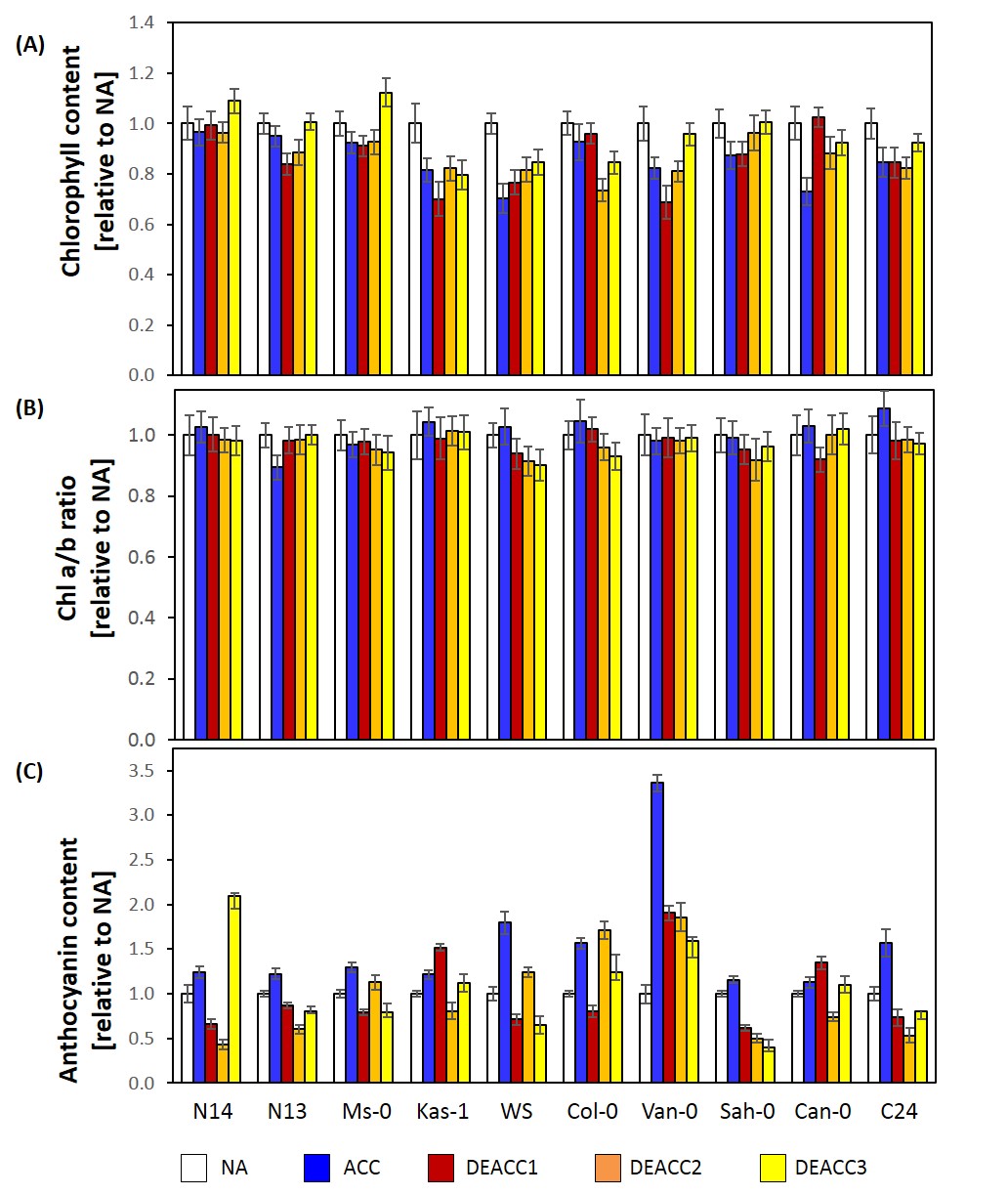

Supplement: Supplementary Figure 5 — Data depicted in Figure 1 normalizes for each accession on the levels of NA plants, Chlorophyll contents (A), Chl a/b ratio (B), and anthocyanin contents (C). Bars represent means ± standard deviation (n = 9). [file Image5.JPEG]
